# Determining core quality of life outcomes for patients with vulvar cancer and their partners: a Delphi consensus study

**DOI:** 10.1007/s00520-026-11270-0

**Published:** 2026-10-06

**Authors:** Astrid Machielsen, Nikki Meijer, Jessy van Dongen, Dorry Boll, Emiel van Haren, Maaike Oonk, Johanna Annemijn W. M. Aarts, Simone Oerlemans, Merel Kimman, Edith van Esch, Nicole Ezendam

**Affiliations:** 1 Department of Obstetrics and Gynecology, Catharina Hospital, Michelangelolaan 2, 5623 EJ Eindhoven, The Netherlands; 2 Faculty of Health, Medicine & Life Sciences, Maastricht University, Universiteitssingel 40, 6229 ER Maastricht, The Netherlands; 3 Department of Plastic and Reconstructive Surgery, Catharina Hospital, Michelangelolaan 2, 5623 EJ Eindhoven, The Netherlands; 4 Department of Obstetrics and Gynecology, University Medical Centre Groningen, University of Groningen, Hanzeplein 1, 9713 GZ Groningen, The Netherlands; 5 Department of Obstetrics and Gynecology, Cancer Center Amsterdam, Amsterdam University Medical Centre, Location VUMC, Meibergdreef 9, 1105 AZ Amsterdam, The Netherlands; 6 Netherlands Comprehensive Cancer Organization, Heidelberglaan 7, 3584 CS Utrecht, The Netherlands; 7 Department of Clinical Epidemiology and Medical Technology Assessment, Care and Public Health Research Institute, Maastricht University Medical Centre, P. Debyelaan 25, 6229 HX Maastricht, The Netherlands; 8 Department of Gynecologic Oncology, Erasmus Medical Center, Erasmus MC Cancer Center, Wytemaweg 80, 3015 CN Rotterdam, The Netherlands; 9 Center of Research On Psychological and Somatic Disorders, Tilburg University, Warandelaan 2, 5037 AB Tilburg, The Netherlands

**Keywords:** Vulvar cancer, Core outcome set, Health-related quality of life, Delphi study

## Abstract

**Purpose:**

This study aimed to develop a patient-centered and partner-centered core outcome set (COS) reflecting the health-related quality of life (HRQoL) outcomes most important to patients with vulvar cancer following surgical treatment and their partners. Identifying these outcomes enables healthcare professionals to better support patients’ HRQoL during and after treatment.

**Methods:**

A mixed-methods study conducted in the Netherlands from December 2021 to March 2024 among patients with vulvar cancer, their partners, and healthcare professionals involved in their care. The study followed a three-step approach: (1) a literature review to identify HRQoL concepts and existing questionnaires; (2) interviews with patients, partners, and healthcare professionals to extract important outcomes; and (3) an online Delphi study, followed by an online consensus meeting, to prioritize outcomes. The primary outcomes were the development of patient-centered and partner-centered COS and the identification of validated questionnaires for HRQoL assessment.

**Results:**

The patient-centered COS comprised 45 outcomes in the domains of physiological functioning, including physical symptoms, as well as physical, sexual, and emotional functioning. The COS also included topics that are less often included in studies, i.e., wound healing, body image, and fear of recurrence. The partner-centered COS included six outcomes only focusing on information provision, not on their own HRQoL. Recommended patient HRQoL questionnaires were (scales from) EORTC QLQ-C30/VU34/SH22/INFO25 and WOUND-Q.

**Conclusions:**

This is the first patient-centered COS for vulvar cancer, enabling targeted HRQoL assessments and improved daily clinical care. The provision of information was the only aspect deemed important as an outcome for partners.

**Supplementary Information:**

The online version contains supplementary material available at https://doi.org/10.1007/s00520-026-11270-0.

## Introduction

Vulvar cancer accounts for about 5% of gynecologic malignancies and is diagnosed more often in developed countries with an increase in incidence over the last decades [1–3]. Treatment is predominantly surgical, ranging from wide local excision with sentinel lymph node dissection, partial or complete vulvectomy with sentinel lymph node dissection or lymphadenectomy, or palliative resection of the tumor [4]. Reconstructive surgery may be used when primary closure is not possible, depending on tumor size or anatomical location. Although surgery has become less radical over the years, it still has a major impact on patients’ health-related quality of life (HRQoL) [5]. Symptoms such as body image concerns, psychosocial challenges, lymphedema, wound issues, pain, and sexual dysfunction are commonly reported by patients and can persist for many years after treatment [5–12].

Patient-reported outcome measures (PROMs) provide valuable insight into patients’ self-reported health status, offering understanding of their symptoms, treatment effects, and overall quality of life (QoL). PROMs support more effective communication between healthcare professionals and patients, enabling personalized care and more informed decision-making [13]. Furthermore, PROMs have been associated with improved symptom management and daily clinical care, especially when PROM results are discussed with the patient [14–17]. To determine which outcomes are most important to measure and discuss in the context of vulvar cancer, it is necessary to develop a core outcome set (COS) that can be applied across clinical practice and research settings. The use of a COS facilitates comparability of findings across studies, reduces reporting bias, and increases the likelihood that outcomes of greatest relevance to patients are consistently assessed and reported [18]. Given the unique and often profound physical, psychosocial, and sexual consequences of vulvar cancer and its treatment, the development of a vulvar cancer-specific COS is warranted. Such COS provides an overview of most relevant constructs that can be measured, preferably using existing well-validated instruments or, if these are not available, newly developed measures.

Cancer also affects partners, whose needs in aftercare remain underexplored while the importance of emotional support and quality of life is positively related in cancer survivors [19]. A partner-centered COS may be necessary to address these needs.

This study aimed to identify outcomes most meaningful to patients with vulvar cancer after surgical treatment and their partners to develop a patient- and partner-centered COS. Identifying these outcomes will enable healthcare professionals to better support the HRQoL of both patients and partners during and after treatment.

## Methods

### Study design

This study was conducted in the Netherlands from December 2021 to March 2024, following a three-step approach: (1) a literature review to identify HRQoL concepts and questionnaires used to assess QoL in vulvar cancer treatment; (2) interviews with patients, partners and healthcare professionals to identify relevant outcomes; and (3) an online patient Delphi study and separate partner Delphi study, comparing their responses with those of healthcare professionals. An online consensus meeting was scheduled to reach final agreement on the outcomes to be included in the patient-centered COS and partner-centered COS. The methodology of this study is in accordance with guidance provided by the Core Outcome Measures in Effectiveness Trials (COMET) Initiative [20, 21].

### Literature review

A literature review was conducted to gain insight in which questionnaires have been used to evaluate QoL in patients with vulvar cancer. The search strategy was designed for MEDLINE (via OvidSP). The search strategy was limited to full-text articles published in English from 2010 to 2021. All search results were exported to Covidence systematic review software (Veritas Health Innovation, Melbourne, Australia, available at www.covidence.org) and screened for eligibility by two researchers (AM, EvE). Articles reporting on or using a questionnaire that evaluated QoL in patients with vulvar cancer were included. The questionnaires that were used as outcome measure in these studies were extracted.

### Interviews with patients, partners and healthcare professionals

The participants involved in this study were patients diagnosed with vulvar cancer within 2 years prior to the start of this study, their partners, and healthcare professionals involved in the management of vulvar cancer (gynecologic oncologists, radiation therapists, oncologists, plastic surgeons, and physician assistants). Patients could participate in case they experienced recurrent disease. For the interviews, participants were recruited via their healthcare professional in Catharina Hospital Eindhoven.

Semi-structured individual interviews, conducted by telephone, were held with patients, partners, and healthcare professionals. The interviews were guided by the commonly used questionnaires identified through the literature review, with additional questionnaires included based on healthcare professionals’ expertise in evaluating HRQoL after vulvar cancer treatment in the Netherlands. Participants were asked about topics and outcomes they considered relevant for assessing HRQoL after surgical treatment for vulvar cancer, as well as any concepts they felt were missing from the presented questionnaires. All interviews were audio-recorded and transcribed verbatim. Two researchers (JvD, NE) analyzed and coded the transcripts using ATLAS.ti, focusing on topics from the presented questionnaires and evaluating their relevance. After coding, thematic analysis was conducted to identify which outcomes and concepts should be carried forward to the Delphi studies. To reduce bias in questioning and interpretation, the interviewer had no prior clinical relationship with the patients and partners.

### Delphi study

For the Delphi studies, patients were recruited by healthcare professionals during consultations in Catharina Hospital Eindhoven, University Medical Center Groningen, University Medical Center Amsterdam, and via the patient organization’s (Stichting Olijf) newsletter and website. Partners of patients were recruited via the patient. Healthcare professionals were recruited by email by the healthcare professionals conducting this study. Written informed consent was obtained from all participants. We aimed to include 30 patients and their partners and 30 healthcare professionals. A two-round online Delphi study was performed between March and November 2023. Participants were asked to judge the importance of the outcomes derived from the interviews on a 10-point Likert scale, from 1 “not important” to 10 “very important.” Patients and partners received a different Delphi survey, only including the outcomes that were extracted from the interviews of the corresponding group. Healthcare professionals participated in both Delphi surveys. The patient and the partner ratings were compared to the healthcare professionals’ ratings. Consensus criteria were defined a priori. If ≥ 70% of participants scored the outcome as “very important” (options 7–10) and < 15% of participants scored the outcome as “not important” (options 1–3), the outcome was considered “consensus in.” Outcomes were considered “consensus out” if ≥ 70% of the participants scored the item as “not important” (options 1–3) and < 15% of participants scored the item as “very important” (options 7–10). All other scoring patterns were taken to indicate “no consensus.”

DelphiManager software, developed by the COMET Initiative, was used to undertake the Delphi surveys. Participants could suggest new outcomes in round 1. At the end of round 1, outcomes that met the definition for “consensus out” were excluded for further consideration, whereas those meeting criteria for “consensus in” were included in the COS. Outcomes that had been determined to be “no consensus” were presented in round 2 with an anonymized graphical summary how the other group rated the outcome and a reminder of the participant’s own score. Participants were given the opportunity to change their score if they wished. After round 2, outcomes considered “very important” by either patients in the patient survey or partners in the partner survey, but < 70% of the healthcare professionals scoring the item as “very important,” were considered “consensus in” based on patient or partner rating to make sure that the outcomes that patients or partners deemed most important were identified.

An online consensus meeting was scheduled to discuss the newly suggested outcomes and the “no consensus” outcomes that persisted after round 2. Outcomes included based on patient or partner ratings were passed forward to the consensus meeting to gain better understanding of the heterogeneity in outcome ratings in comparison to healthcare professionals, but were not voted on to make sure that the outcomes deemed important by patients or partners are included in the patient-centered and partner-centered COS.

### Questionnaire selection

The overview of QoL questionnaires obtained from this study through literature review, to interviews and Delphi consensus meeting, were used to identify which questionnaires best cover the outcomes included in the patient-centered and partner-centered COS. This process will inform our recommendation on the most suitable questionnaire or measurement instrument for assessment of the patient-centered and partner-centered COS.

## Results

### Literature review

The full search strategy is shown in the Supporting Information (Appendix S1). In total, 301 records were identified. After title and abstract screening, 47 records were evaluated in full-text and 46 articles met eligibility criteria. The included studies and extracted data are presented in the Supporting Information (Figure S1; Table S1). QoL was assessed in 31 articles (65.9%). A total of 46 different questionnaires, excluding self-developed questionnaires, were used to evaluate QoL. The questionnaires most commonly used were Female Sexual Function Index (FSFI) (*n* = 10), European Organization for Research and Treatment Quality of Life Questionnaire Core 30 (EORTC QLQ-C30) (*n* = 10), Functional Assessment of Cancer Therapy-Vulva (FACT-V) (n = 6), Functional Assessment of Cancer Therapy-General (FACT-G) (*n *= 4), and 12-Item Short Form Health Survey (SF-12) (*n* = 4).

### Interviews with patients, partners, and healthcare professionals

In total, 28 interviews were conducted with 10 patients, 8 partners, and 10 healthcare professionals (Table 1). The EORTC QLQ-C30, European Organization for Research and Treatment Quality of Life Questionnaire vulva cancer module (EORTC QLQ-VU34), WOUND-Q, Body Image Scale (BIS), European Organization for Research and Treatment Sexual Health Questionnaire (EORTC SHQ-C22), FSFI, Female Sexual Distress Scale (FSDS), and Maudsley Marital Questionnaire (MMQ) were presented to patients based on the results of the literature review. The MMQ, EORTC SHQ-C22, Hospital Anxiety and Depression Scale (HADS), Dyadic Coping Inventory (DCI), and Cancer Survivors’ Unmet Needs (CaSUN) were presented to partners. All questionnaires were presented to the healthcare professionals.

**Table 1:** Participant characteristics interviews

Patients (*n* = 10)	
**Age (years)**	Mean age 64,5 years (range 44–85 years)
**Partner**	9 (90%
**Time since first diagnosis (years)**	
1–3	3 (30%)
4–6	4 (40%)
≥ 7	3 (30%)
**Recurrence**	3 (30%)
**FIGO staging**	
I and II	7 (70%)
III	3 (30%)
**Lichen sclerosus**	8 (80%)
**Treatment**	
Surgery	10 (100%)
Adjuvant radiotherapy	2 (20%)
**Lymph node dissection**	
Sentinel node	5 (50%)
Unilateral	1 (20%)
Bilateral	4 (80%)
Lymphadenectomy	4 (40%)
Unilateral	2 (50%)
Bilateral	2 (50%)
No lymph nodes examined	1 (10%)
**Clitoral resection**	2 (20%)
**Urethral involvement**	4 (40%)
**Anal sphincter involvement**	2 (20%)
**Plastic reconstruction**	5 (50%)
**Partners (*n* = 8)**	
**Healthcare professionals (*n* = 10)**	
Gynecologic oncologist	5 (50%)
Plastic surgeon	3 (30%)
Physician assistant	2 (20%)

### Comments on the questionnaires presented to the patients and healthcare professionals

Patients and healthcare professionals were both interested in outcomes related to daily functioning, such as the ability to work, to perform sports (e.g., cycling), and the impact on wearing clothes. The patients’ supportive care needs were perceived relevant to healthcare professionals and a missing concept in the questionnaires presented to the patients. Physical symptoms seemed to be of less importance, except for pain, wound-related symptoms, urinary and fecal incontinence, and lymphedema. Emotional well-being, in particular fear of recurrence and body image, was an important concept for both patients and healthcare professionals. Although sexual functioning was important to almost all patients and healthcare professionals, this was not always discussed during their treatment course and patients wished to get support with problems related to sexuality. The outcomes related to sexuality that were important to both patients and healthcare professionals were problems related to lubrication, difficulties in achieving orgasm, pain, and fear of resumption of sexual activities. Both patients and healthcare professionals mentioned that sexuality starts with feeling comfortable with looking at and touching their own genital area, a missing concept in the questionnaires presented to the patients. Furthermore, relational functioning was discussed, with patients mentioning feelings of embarrassment and inferiority towards partner, the importance of communication, and coping with relationship stress. Satisfaction about information provision was a missing concept in the questionnaires. Therefore, concepts from the EORTC QLQ-INFO25 were included in the Delphi survey. In total, 55 patient outcomes were extracted and included in the patient Delphi study (Table S2).

### Comments on the questionnaires presented to the partners and healthcare professionals

All partners and the majority of the healthcare professionals emphasized the importance of giving attention to sexuality and communication in the relationship. Outcomes related to sexuality of importance to partners were worrying about their partner’s sexual pleasure and worrying about hurting their partner during sexual activities. However, the majority of the healthcare professionals was less interested in the assessment of the partner’s well-being because “daily clinical care should be patient-centered.” The healthcare professionals also claimed that this would “raise expectations of treating the partner as well.” The majority of the partners confirmed that the patient should be the primary focus. Information provision was also important to partners. Therefore, concepts from the EORTC QLQ-INFO25 were included in the Delphi survey. In total, 23 partner outcomes were extracted and passed forward to the partner Delphi study (Table S3).

#### Delphi study

The 78 outcomes derived from the literature review and interviews were mapped to domains within the COMET taxonomy to better understand the spread of outcomes [20]. The COMET taxonomy was expanded by adding the domain “functioning – sexual functioning.” The results of the Delphi studies are shown in the Supporting Information (Table S2-S6).

A total of 70 participants registered for round 1, including 34 patients, 12 partners, and 24 healthcare professionals (Table 2). There were 54 participants in round 2, including 27 patients (20,6% attrition), 9 partners (25% attrition), and 18 healthcare professionals (25% attrition).

**Table 2:** Participant characteristics Delphi study and consensus meeting

Patients		
	Delphi study (***n*** = 34)	Consensus meeting (***n*** = 8)
**Age (years)**	Mean age 60.5 years (range 30–91)	Mean age 57.1 years (range 36–76)
**Partner**	27 (79.4%)	6 (75%)
**Time since first diagnosis (years)**		
1	9 (26.5%)	4 (50%)
2	13 (38.2%)	3 (37.5%)
≥ 3	12 (35.3%)	1 (12.5%)
**Recurrence**	3 (8.8%)	1 (12.5%)
**FIGO staging**		
I and II	17 (50%)	4 (50%)
III	1 (2.9%)	1 (12.5%)
IV	3 (8.8%)	0
Unknown	13 (38.2)	3 (37.5%)
**Lichen sclerosus**	18 (52.9%)	5 (62.5%)
**Treatment**		
Surgery	34 (100%)	8 (100%)
Adjuvant radiotherapy	4 (11.8%)	2 (25%)
**Lymph node dissection**		
Sentinel node	27 (79.4%)	8 (100%)
Unilateral	3 (8.8%)	0
Bilateral	24 (70.6%)	8 (100%)
Lymphadenectomy	5 (14.7%)	0
Unilateral	3 (8.8%)	
Bilateral	2 (5.9%)	
Unknown	2 (5.9%)	0
**Clitoral resection**	12 (35.3%)	4 (50%)
**Urethral involvement**	6 (17.7%)	3 (37.5%)
**Anal sphincter involvement**	3 (8.8%)	0
**Plastic reconstruction**	9 (26.5%)	3 (37.5%)
**Partners**		
	**Delphi study (*n* = 12)**	
**Age (years)**	Mean age 58.5 years (range 49–73 years)	
**Healthcare professionals**		
	**Delphi study (*n* = 24)**	**Consensus meeting (*n* = 5)**
Gynecologic oncologist	12	4
Plastic surgeon	3	0
Oncologist	1	1
Radiation therapist	4	0
Physician assistant	4	0

### Delphi study patients

The first patient survey included 55 outcomes. After round 1, 28 outcomes reached “consensus in,” 5 were “consensus out,” and 22 remained “no consensus.” Heterogeneity in ratings primarily involved the domains of “sexual functioning” and “delivery of care.” Four new outcomes were suggested: “removal of (a part of) the clitoris,” “vaginal odor,” “change in genital sensation,” and “impact on entering (new) relationships.” The 22 “no consensus” outcomes advanced to round 2.

In round 2, 2 additional outcomes were rated as “consensus in” and 11 outcomes were included based on patient ratings. Nine outcomes remained “no consensus” and were forwarded to the consensus meeting. After both rounds, 39 outcomes were “consensus in,” 11 were added based on patient ratings, and 6 were excluded as “consensus out.” The consensus meeting reviewed the remaining 9 “no consensus” outcomes and 4 newly suggested outcomes.

### Delphi study partners

The partner survey began with 23 outcomes. After round 1, none were “consensus in,” 11 were “consensus out,” and 12 remained “no consensus.” Again, heterogeneity in ratings primarily involved the domains of “sexual functioning” and “delivery of care.” The 12 “no consensus” outcomes advanced to round 2. In round 2, 2 outcomes were rated as “consensus out,” 2 as “consensus in,” and 4 were included based on partner ratings—all within the domain “delivery of care.”

A planned consensus meeting for 4 “no consensus” outcomes was canceled due to low partner participation, with partners emphasizing patient-centered care. Ultimately, 2 outcomes were “consensus in” and 4 more were included based on partner ratings. The final partner-centered COS contained 6 outcomes (Table 3), which could be assessed using the EORTC QLQ-INFO25 questionnaire, though partners noted limited value in additional questionnaires during aftercare.

**Table 3:** Partner-centered COS

Domain	Outcomes
Delivery of care	Information provision; information about disease/treatment/medical examinations/practical issues; satisfaction with information provision

### Consensus meeting with patients and healthcare professionals

Thirteen participants (8 patients, 5 healthcare professionals) attended an online consensus meeting (Table 2). Outcomes were categorized by domain and reviewed. Most “no consensus” outcomes related to “sexual functioning.” Patients noted that while sexuality is important, detailed discussions are not always necessary but should be revisited periodically as needs may change. Additionally, patients emphasized the need for tailored, timely information throughout their treatment course.

During the meeting, 8 outcomes were voted “consensus in,” and 4 were “consensus out.” Four outcomes related to fear of recurrence were consolidated into a single outcome due to their interrelation. The final patient-centered COS included 45 outcomes (Table 4).

**Table 4:** Patient-centered COS and proposed questionnaire (scales) that can be used to assess the outcome

Domain	Outcomes	Questionnaire scale(s) and/or item(s) from item bank
**Physiological** Outcomes relating to neoplasms	Wound healing; wound management; vulvar skin changes; scar tissue; vaginal swelling; lymphedema groin area; lymphedema legs; urinary problems; urinary incontinence; fecal incontinence; lichen sclerosus^a^; vulvar changes related to sexuality; vaginal odor; change in sensation of the genital area	WOUND-Q scale “assessment”/“dressing”; EORTC QLQ-VU34* scale “vulva skin changes”/“vulva scarring”/“vulva swelling”/“groin lymphedema”/“leg lymphedema”/“urine urgency and leakage”/“bowel urgency and leakage”/“sexually related vaginal changes”; FACT-V additional concerns V2/V6
**Functioning** Physical functioning	Role functioning; physical functioning; cycling^a^; sitting; wearing clothes; wearing pads or incontinence material	EORTC QLQ-C30* scale “role functioning”/“physical functioning”; EORTC QLQ-VU34 scale “vulva skin changes”; EORTC Q273/Q52
**Functioning** Sexual functioning	Sexual functioning; pain during intercourse; communication about sexuality; sexual satisfaction; desire to be sexually active	EORTC QLQ-SH22* scale “sexual satisfaction”/“communication scale”; EORTC-VU34 scale “sexual enjoyment”
**Functioning** Social functioning	Social functioning; relationship satisfaction	EORTC QLQ-C30; EORTC Q29
**Functioning** Emotional functioning	Impact of wound on mood; emotional functioning; body image; feeling comfortable with genital area^a^; concerns about family; intimacy^a^; intimacy satisfaction; fear of recurrence; confidence in body	WOUND-Q, scale “psychological”; EORTC QLQ-C30; EORTC QLQ-VU34 scale “body image”; EORTC Q167/Q364/Q415; EORTC QLQ-SH22 scale “sexual satisfaction”
**Functioning** Cognitive functioning	Cognitive functioning	EORTC QLQ-C30 scale “cognitive functioning”
**Global quality of life**	Quality of life; impact of wound on quality of life^a^	EORTC QLQ-C30 scale “global health status/QoL”
**Delivery of care**	Information provision; information about disease/treatment/medical examinations/practical issues; satisfaction with information provision	EORTC QLQ-INFO25*

^a^No questionnaire available

^*^Abbreviations: *EORTC QLQ-VU34*, European Organization for Research and Treatment Quality of Life Questionnaire vulva cancer module; *FACT-V*, Functional Assessment of Cancer Therapy – Vulva; *EORTC QLQ-C30*, European Organization for Research and Treatment Quality of Life Questionnaire Core 30; *EORTC QLQ-SH22*, European Organization for Research and Treatment Quality of Life Questionnaire Sexual Health; *EORTC QLQ-INFO25*, European Organization for Research and Treatment Quality of Life Questionnaire INFO25

#### Questionnaire selection

The (scales of the) EORTC QLQ-C30/VU34/SH22/INFO25, FACT-V and WOUND-Q questionnaires collectively cover almost all outcomes included in the patient-centered COS. Table 4 provides an overview of the patient-centered COS and details on how these outcomes can be measured.

There are no questionnaires available that measure outcomes such as “lichen sclerosus,” “cycling,” “wearing clothes,” “feeling comfortable with genital area,” “intimacy,” and “impact of wound on QoL.” However, during the consensus meeting, patients reported that lichen sclerosus is related to fear of recurrence and this could be measured by using the EORTC Q364 from the EORTC item bank or the Fear of Cancer Recurrence Inventory (FCRI). In addition, “wearing clothes” is perceived important to patients because of the inability to wear tight clothes after surgery and feelings of embarrassment because of complications such as lymphedema. The inability to wear tight clothes could be measured by using the EORTC Q273 from the EORTC item bank. Communication about sexuality was important to patients and was primarily focused on discussion with partners. However, during the consensus meeting, it became clear that it was also important for patients to talk about sexuality with their healthcare professionals. Therefore, both the EORTC QLQ-SH22 scale “sexual satisfaction” and the “communication scale” are recommended, asking explicitly if patients talked about sexuality with the healthcare professional. The outcome “relationship satisfaction” could be measured by using the EORTC Q29 from the EORTC item bank; however, this outcome does not specifically measure the interaction between the patient and partner but is broader. If the interaction between the patient and partner needs to be explored in more depth, the Maudsley Marital Questionnaire is recommended. Intimacy could not be measured by existing measurement instruments; however, intimacy satisfaction is also included in the patient-centered COS and could be measured by using the EORTC QLQ-SH22 scale “sexual satisfaction.”

## Discussion

This is the first study to describe the development of a patient-centered COS specifically addressing the challenges faced by patients with vulvar cancer and their partners. A patient-centered core outcome set (COS) was developed, consisting of 45 outcomes that primarily cover the domains of “physiological” functioning, including physical symptoms, as well as physical, sexual, and emotional functioning. There is no need for a partner-centered COS.

Patients highlighted several domains of HRQoL as important, underscoring the multifaceted nature of HRQoL and emphasizing the necessity of assessing outcomes beyond physical health status alone. Including patients’ perspectives in HRQoL assessment is crucial for delivering patient-centered care and addressing specific needs effectively. By understanding and incorporating these aspects into clinical practice, healthcare professionals can tailor interventions and support strategies to enhance the overall well-being and satisfaction of patients following treatment for vulvar cancer.

The only outcomes deemed important by partners and subsequently included in the partner-centered COS were related to information provision. Partners emphasized the importance of maintaining a patient-centered focus and noted that an additional questionnaire for partners during aftercare offered limited meaningful value. This highlights the need to enhance patient-centered care while addressing partners’ perspectives, particularly in terms of information provision.

This study provides novel insights into the development of a patient-centered COS, addressing critical gaps in the literature on HRQoL outcomes for patients with vulvar cancer. The findings align with previous research emphasizing the importance of incorporating patient perspectives into outcome measurement to deliver tailored, patient-centered care [22]. By prioritizing the domains of HRQoL identified as significant by patients, this study reaffirms the multidimensional nature of well-being and the need to move beyond purely clinical outcomes. Importantly, the outcomes in the COS can for the most part be measured with scales/items from existing validated questionnaires: the EORTC QLQ-C30/VU34/SH22/INFO25, FACT-V [23], and WOUND-Q. A combination of items and scales from all questionnaires allows for the measurement of the COS outcomes. All EORTC questionnaires have a similar answering format that facilitates designing the final instrument.

The limited inclusion of partner-reported outcomes, focusing primarily on information provision, aligns with prior evidence highlighting partners’ roles in supporting patients while advocating for patient-centered care. However, the finding that additional questionnaires for partners add limited value to aftercare is a critical consideration for future research and clinical practice.

This study’s systematic approach and adherence to established guidelines strengthen the credibility of its findings, and the use its systematic approach, which incorporated mixed-methods such as a literature review and qualitative interviews to inform the development of a comprehensive longlist of outcomes. This ensured that the outcomes included in the Delphi study were both relevant and robust. The study’s adherence to the COMET guidelines for COS development further supports the validity and reproducibility of its findings. The inclusion of multiple rounds of feedback in the Delphi study allowed for the integration of diverse insights and enhanced the comprehensiveness of the COS. Anonymity in responses also minimized biases associated with hierarchical relationships among participants.

However, several limitations should be acknowledged. The relatively small sample size of patients and partners in the Delphi study, although reflective of the low incidence of vulvar cancer, may have influenced the identified outcomes and limited the generalizability of the findings. Furthermore, the iterative nature of the Delphi method, while promoting consensus, is time-consuming and can lead to attrition. In this study, a relatively high attrition rate was observed, which may have reduced the diversity of perspectives contributing to the final COS. Another limitation lies in the absence of a single comprehensive questionnaire capable of measuring all concepts included in the patient-centered COS. Existing scales and items from item libraries must be combined to address all outcomes comprehensively. While many outcomes in the COS can be measured using the EORTC QLQ-VU34, this questionnaire is now in phase 4 of development and validation and is developed to use in clinical trials [24].

The challenges identified, such as the need for comprehensive measurement tools and strategies to mitigate attrition in Delphi studies, are consistent with challenges noted in similar COS development studies. Future work must focus on the practical implementation of this COS in clinical settings and explore methodologies for routine assessment. The patient-centered COS developed through this study will be integrated into the IMPACT-V project in the Netherlands, a quality improvement initiative for vulvar cancer care. This integration will enable ongoing evaluation of HRQoL outcomes, ensuring that care remains patient-centered and responsive to the needs of patients and their partners.

## Conclusions

This study resulted in a patient-centered COS comprising 45 outcomes deemed important by patients for assessment during the aftercare following surgical treatment for vulvar cancer. These outcomes spanned a range of domains on physiological, physical, sexual, social, emotional, and cognitive functioning, global quality of life, and information provision. The findings provide valuable insights into the outcomes that matter most to patients with vulvar cancer and highlight the necessity of incorporating these outcomes into aftercare assessments. From the partners’ perspective, the partner-centered COS only includes outcomes related to information provision, suggesting that partners prioritize the delivery of information.

## Supplementary Information

Below is the link to the electronic supplementary material.

Supplementary file1 (DOCX 76.1 KB)

## Data Availability

The data that supports the findings of this study are available in the supplementary material of this article. Additional information is available from the corresponding author upon request.
