# Supplementary material for: Determining core quality of life outcomes for patients with vulvar cancer and their partners: a Delphi consensus study
Source: Support Care Cancer. 2026 Oct 6;34(10):1061. doi: 10.1007/s00520-026-11270-0 (PMC13642464; doi:10.1007/s00520-026-11270-0)
Supplement: Supplementary file 1 [file 520_2026_11270_MOESM1_ESM.docx]

**Appendix S1: Search strategy**

Search: (((vulvar cancer[Title/Abstract]) OR (vulvar neoplasms[Title/Abstract])) OR (carcinoma of the vulva[Title/Abstract])) AND ((((((((((((((questionnaire) OR (tool)) OR (instrument)) OR (scale)) OR (survey)) OR (interview)) OR ("qualitative study"[All Fields])) OR ("focus group"[All Fields])) OR (outcome measure)) OR (outcome assessment)) OR ("patient reported outcome"[All Fields])) OR (PROM)) OR ("patient reported outcome measure"[All Fields])) OR ((((quality of life) OR (QOL)) OR ("health related quality of life"[All Fields])) OR (HRQOL))) Filters: Full text, English, from 2010 - 2021

(("vulvar cancer"[Title/Abstract] OR "vulvar neoplasms"[Title/Abstract] OR "carcinoma of the vulva"[Title/Abstract]) AND ("questionnair"[All Fields] OR "questionnaire s"[All Fields] OR "surveys and questionnaires"[MeSH Terms] OR ("surveys"[All Fields] AND "questionnaires"[All Fields]) OR "surveys and questionnaires"[All Fields] OR "questionnaire"[All Fields] OR "questionnaires"[All Fields] OR "tool"[All Fields] OR ("instrument"[All Fields] OR "instrument s"[All Fields] OR "instrumentation"[MeSH Subheading] OR "instrumentation"[All Fields] OR "instruments"[All Fields] OR "instrumented"[All Fields] OR "instrumenting"[All Fields]) OR ("scale s"[All Fields] OR "scaled"[All Fields] OR "scaling"[All Fields] OR "scalings"[All Fields] OR "weights and measures"[MeSH Terms] OR ("weights"[All Fields] AND "measures"[All Fields]) OR "weights and measures"[All Fields] OR "scale"[All Fields] OR "scales"[All Fields]) OR ("survey s"[All Fields] OR "surveyed"[All Fields] OR "surveying"[All Fields] OR "surveys and questionnaires"[MeSH Terms] OR ("surveys"[All Fields] AND "questionnaires"[All Fields]) OR "surveys and questionnaires"[All Fields] OR "survey"[All Fields] OR "surveys"[All Fields]) OR ("interview"[Publication Type] OR "interviews as topic"[MeSH Terms] OR "interview"[All Fields]) OR "qualitative study"[All Fields] OR "focus group"[All Fields] OR ("outcome assessment, health care"[MeSH Terms] OR ("outcome"[All Fields] AND "assessment"[All Fields] AND "health"[All Fields] AND "care"[All Fields]) OR "health care outcome assessment"[All Fields] OR ("outcome"[All Fields] AND "measure"[All Fields]) OR "outcome measure"[All Fields]) OR ("outcome assessment, health care"[MeSH Terms] OR ("outcome"[All Fields] AND "assessment"[All Fields] AND "health"[All Fields] AND "care"[All Fields]) OR "health care outcome assessment"[All Fields] OR ("outcome"[All Fields] AND "assessment"[All Fields]) OR "outcome assessment"[All Fields]) OR "patient reported outcome"[All Fields] OR ("patient relat outcome meas"[Journal] OR "prom"[All Fields]) OR "patient reported outcome measure"[All Fields] OR ("quality of life"[MeSH Terms] OR ("quality"[All Fields] AND "life"[All Fields]) OR "quality of life"[All Fields] OR "QOL"[All Fields] OR "health related quality of life"[All Fields] OR ("hrqols"[All Fields] OR "quality of life"[MeSH Terms] OR ("quality"[All Fields] AND "life"[All Fields]) OR "quality of life"[All Fields] OR "hrqol"[All Fields])))) AND ((fft[Filter]) AND (english[Filter]) AND (2010:2021[pdat]))

Figure S1: Study selection process for literature review

**Table S1: Overview literature review**

| **Title** | **Author(s)** | **Publication year** | **Quality of Life (QoL) assessment** |
| --- | --- | --- | --- |
| Impact of radical operative treatment on the quality of life in women with vulvar cancer - a retrospective study | Günther, V. et al. | 2014 | EORTC QLQ-C30*; HSI*; EORTC supplementary questions including questions concerning the emotional burden of treatment and confidence in the future; vulva-specific module developed by Küchler and Malchow. |
| Long-term sexual function in survivors of vulvar cancer: a cross-sectional study | Hazewinkel, M.H. et al. | 2012 | FSFI*; SF-36*; BIS*; LOT*. |
| Body mass index and sexual function in women with gynaecological cancer | Donkers, H. et al. | 2019 | FSFI. |
| Outcomes after radiation therapy with concurrent weekly platinum-based chemotherapy or every-3-4-week 5-fluorouracil-containing regimens for squamous cell carcinoma of the vulva | Mak, R.H. et al. | 2011 | No data on QoL reported. |
| Radiotherapy versus inguinofemoral lymphadenectomy as treatment for vulvar cancer patients with micrometastases in the sentinel node: results of GROINSS-V II | Oonk, M.H.M. et al. | 2021 | No data on QoL reported. |
| Versatility of pedicled anterolateral thigh flap in gynecologic reconstruction after vulvar cancer extirpative surgery | Gentileschi, S. et al. | 2017 | No data on QoL reported. |
| Chemoradiation for advanced primary vulval cancer | Shylasree, T.S. et al. | 2011 | No data on QoL reported. |
| Profunda artery perforator flap for isolated vulvar defect reconstruction after oncological resection | Chang, T.N. et al. | 2016 | FACT-G*; FACT-V*. |
| A patient-reported outcome measure to identify occurrence and distress of post-surgery symptoms of WOMen with vulvAr Neoplasia (WOMAN-PRO) - a cross sectional study | Senn, B. et al. | 2013 | WOMAN-PRO instrument. |
| A modified triple incision technique for women with locally advanced vulvar cancer: a description of the technique and outcomes | Li, J. et al. | 2012 | No data on QoL reported. |
| Quality of life and sexuality of patients after treatment for gynaecological malignancies: results of a prospective study in 55 patients | Pilger, A. et al. | 2012 | FSFI; SF-12*; self-developed questionnaire including demographic features and questions about symptoms, frequency of sexual activities, sources of information about sexuality, and barriers of communication about sexual problems. |
| Feasibility, indications and complications of SCIP flap for reconstruction after extirpative surgery for vulvar cancer | Gentileschi, S. et al. | 2022 | No data on QoL reported. |
| Comparison of two types of the triple incision technique in the treatment of patients with locally advanced vulvar cancer | Ma, Y. et al. | 2020 | No data on QoL reported. |
| Adjuvant radiotherapy and local recurrence in vulvar cancer - a subset analysis of the AGO-CaRE-1 study | Woelber, L. et al. | 2022 | No data on QoL reported. |
| GOG 244 - The Lymphedema and Gynecologic cancer (LeG) study: the impact of lower-extremity lymphedema on quality of life, psychological adjustment, physical disability, and function. | Carter, J. et al. | 2021 | FACT-G; FACT-V and items from other FACT disease-specific modules; several items from PROMIS SexFS*; IES*; LEFS*; GCLQ*. |
| Outcome of vulvar reconstruction by anterolateral thigh flap in patients with advanced and recurrent vulvar malignancy | Zhang, W. et al. | 2015 | Involved assessment of pain and performance status, using respectively VRS-4* and ECOG Performance Status Scale*. |
| Safety and feasibility of single-incision radical vulvectomy: a novel approach for the treatment of vulvar cancer | He, L. et al. | 2021 | No data on QoL reported. |
| Patient reported outcome measures among patients with vulvar cancer at various stages of treatment, recurrence, and survivorship | Alimena, S. et al. | 2021 | EORTC QLQ-C30; PROMIS-ES*; FACT-V. |
| Quality of life in women with vulvar cancer submitted to surgical treatment: a comparative study | De Melo Ferreira, A.P. et al. | 2012 | EORTC QLQ-C30; ICIQ-UI SF*; FSFI. |
| Quality of life (QOL) in women treated for gynecologic malignancies with radiation therapy: a literature review of patient-reported outcomes | Mirabeau-Beale, K.L. et al. | 2014 | Literature review that summarized currently existing QoL measurement instruments: EORTC QLQ-C30; QOL-CS*; EQ-5D*; SF-36; FACT-G; SAQ*; CALGB SF*; ACOG Sexual Symptom Checklist*; FSFI; PROMIS SexFS. |
| Clinical application of sartorius tendon transposition during radical vulvectomy: a case control study of 58 cases at a single institution | Li, L. et al. | 2015 | EORTC QLQ-C30. |
| The impact of vulvar cancer on psychosocial and sexual functioning: a literature review | Malandrone, F. et al. | 2021 | Literature review that summarized biological, psychological and social factors that impact psychosocial and sexual functioning, including: type and extent of surgery (and removal of the clitoris), genital changes, lymphedema, fatigue, weakness and tiredness, wound-related symptoms (including pain, tingling, swelling, postoperative complications), low self-esteem (including negative body image), sexual dysfunction (including reduced sexual desire, reduced satisfaction, reduced sexual activity, difficulty in lubrication and achieving orgasm, avoidance behavior, and avoiding touching genital area), fear (associated with pain on resumption of sexual intercourse, treatment, and illness progression), evoked emotions (including anxiety, depression, emotional distress (provoked by uncertainty), shame, and embarrassment), negatively affected interpersonal interactions (including social stigma, social isolation, reluctance to initiate a relationship, decrease in relationship satisfaction), difficulties in performing daily activities (including self-care, reduced ability to work and to perform household duties), older age, and poor overall well-being. |
| Can clitoris-conserving surgery for early vulvar cancer improve the outcome in terms of quality of life and sexual sensation? | Forner, D.M. et al. | 2013 | FSFI; SF-12. |
| Outcome of vulvar reconstruction in patients with advanced and recurrent vulvar malignancies | Zhang, W. et al. | 2015 | Involved assessment of pain and performance status, using respectively VRS-4 and ECOG Performance Status Scale. |
| GOG 244 - The LymphEdema and Gynecologic cancer (LEG) study: the association between the gynecologic cancer lymphedema questionnaire (GCLQ) and lymphedema of the lower extremity (LLE) | Carter, J. et al. | 2019 | GCLQ; LEFS. |
| Quality of life and associated factors after surgical treatment of vulvar cancer by vulvar field resection (VFR) | Trott, S. et al. | 2020 | Patient interviews including questions about postoperative morbidity; EORTC QLQ-C30; GCLQ; self-developed questionnaire including items about sexuality. |
| High-dose intensity-modulated chemoradiotherapy in vulvar squamous cell carcinoma: outcome and toxicity | Rishi, A. et al. | 2020 | No data on QoL reported. |
| Screening for distress in women with gynaecological cancer: adaptation of the distress thermometer for gynaecological oncology patients | Harbeck, E. et al. | 2021 | Distress Thermometer-Gynaecological Oncology. |
| Outcomes of Bleomycin-based electrochemotherapy in patients with repeated loco-regional recurrences of vulvar cancer | Pellegrino, A. et al. | 2016 | FACT-V. |
| Quality of life and sexual function after surgery in early stage vulvar cancer | Forner, D.M. et al. | 2015 | FSFI; SF-12. |
| Sexuality and body image following treatment for early-stage vulvar cancer: a qualitative study | Barlow, E.L. et al. | 2014 | Patient interviews that gained insight in important factors related to sexuality and body image, including: fear (associated with: (potential) removal of, or disruption to, the clitoris, pain, and the potential for disruption to sexual intercourse), changes in sensation (including numbness, difficulty in achieving orgasm, and avoiding touching parts of the genital area), sexual relationship (including reluctance to initiate a (sexual) relationship), lack of visibility of the vulva and lymphedema (including having to change style of dress to cover up the lymphedema, worry about developing lymphedema, and adhering to preventative techniques). |
| Sexual activity and function after surgical treatment in patients with (pre)invasive vulvar lesions | Grimm, D. et al. | 2016 | FSFI. |
| Vulvar reconstruction by a ‘supra-fascial’ lotus petal flap after surgery for malignancies | Bodin, F. et al. | 2012 | No data on QoL reported. |
| Vulvar reconstruction following surgery for vulvar cancer using a stepladder V-Y advancement medial thigh flap | Nomura, H. et al. | 2015 | No data on QoL reported. |
| Quality of life after complete lymphadenectomy for vulvar cancer: do women prefer sentinel lymph node biopsy? | Farrell, R. et al. | 2014 | Patient interviews including questions about postoperative morbidity; linear graph to mark the effect the removal of lymph nodes had on their overall QoL; UBC-Q*. |
| Sexual morbidity associated with poorer psychological adjustment among gynecological cancer survivors | Levin, A.O. et al. | 2010 | Questionnaire including the FSFI and several items from the FACT-V and DSFI*; CES-D*; PCL-C*; IES-R*; ITS*; SF-12; FSI TDI*. |
| Sexual, psychological, and relational functioning in women after surgical treatment for vulvar malignancy: a literature review | Aerts, L. et al. | 2012 | Literature review that summarized factors that impact sexual functioning, relational functioning, and overall QoL including: itching, vaginal discharge, sexual dysfunction (including decrease of sexual desire, difficulties with arousal and orgasm, decrease in sexual satisfaction), decrease in relationship satisfaction (including worsened emotional relationship), emotional distress (including mood disturbance and depression), negative body image (including decrease in self-confidence and decrease in body acceptance), tiredness, older age, type and extent of surgery, and poor overall well-being.  And that summarized currently existing QoL measurement instruments: IBCS*; self-developed Genital Sensation List; self-developed questionnaire(s); DSFI; Heterosexual Behavior Hierarchy; SAI*; Groningen Arousability Scale; GSI*; KAS*; BDI*; PRIME-MD PQ*; Kellner’s Symptom Questionnaire; POMS*; MMPI*; HADS*; FSFI; EORTC QLQ-C30; GOG PS*. |
| Primary versus preoperative radiation for locally advanced vulvar cancer | Natesan, D. et al. | 2017 | No data on QoL reported. |
| Modified gluteal fold V-Y advancement flap for vulvovaginal reconstruction | Lee, J.H. et al. | 2013 | No data on QoL reported. |
| Quality of life in women with gynecologic cancer in Turkey | Goker, A. et al. | 2011 | EORTC QLQ-C30. |
| Perceived health-related quality of life in women with vulvar neoplasia: A cross sectional study | Kobleder, A. et al. | 2016 | SF-36; WOMAN-PRO instrument. |
| Sexual activity and function in patients with gynecological malignancies after completed treatment | Grimm, D. et al. | 2015 | Questionnaire including the SAQ and several items of the FSFI and EORTC QLQ-C30. |
| A prospective study in the evaluation of quality of life after vulvar cancer surgery | Novackova, M. et al. | 2015 | EORTC QLQ-C30. |
| A comparative study of video endoscopic inguinal lymphadenectomy and conventional open inguinal lymphadenectomy for treating vulvar cancer | Zhang, M. et al. | 2017 | FACT-G; FACT-V; BIQ*. |
| A prospective study in detection of lower-limb lymphedema and evaluation of quality of life after vulvar cancer surgery | Novackova, M. et al. | 2012 | EORTC QLQ-C30. |
| Multidisciplinary personalized approach in the management of vulvar cancer - the Vul.Can Team experience | Tagliaferri, L. et al. | 2020 | No data on QoL reported. |

***Abbreviations:** EORTC QLQ-C30: European Organization for Research and Treatment Quality of Life Questionnaire Core 30; HSI: Hornheider Screening Instrument; FSFI: Female Sexual Function Index; SF-36: 36-Item Short Form Health Survey; BIS: Body Image Scale; LOT: Life Orientation Test; FACT-G: Functional Assessment of Cancer Therapy - General; FACT-V: Functional Assessment of Cancer Therapy - Vulva; SF-12: 12-Item Short Form Health Survey; PROMIS SexFS: Patient-Reported Outcomes Measurement Information System Sexual Function and Satisfaction measure; IES: Impact of Event Scale; LEFS: Lower Extremity Functional Scale; GCLQ: Gynecologic Cancer Lymphedema Questionnaire; VRS-4: Four-category Verbal Rating Scale; ECOG Performance Status Scale: Eastern Cooperative Oncology Group Performance Status Scale; PROMIS-ES: Patient-Reported Outcomes Measurement Information System Emotional Support measure; ICIQ-UI SF: International Consultation on Incontinence Questionnaire-Urinary Incontinence Short Form; QOL-CS: Quality of Life - Cancer Survivor; EQ-5D: EuroQol-5D; SAQ: Sexual Activity Questionnaire; CALGB SF: Cancer and Leukemia Group B Sexual Functioning Scale; ACOG Sexual Symptom Checklist: American College of Obstetricians and Gynecologists Sexual Symptom Checklist; UBC-Q: Utility-Based-Questionnaire-Cancer; DSFI: Derogatis Sexual Functioning Inventory; CES-D: Center for Epidemiologic Studies Depression scale; PCL-C: PTSD Checklist Civilian Version; IES-R: Impact of Event Scale - Revised; ITS: Impact of Treatment Scale; FSI TDI: Total Disruption Index of the Fatigue Symptom Inventory; IBCS: Intimate Bodily Contact Scale of Vennix; SAI: Sexual Arousability Index; GSI: Global Severity Index; KAS: Katz Adjustments Scales; BDI: Beck Depression Inventory; PRIME-MD PQ: Primary Care Evaluation of Mental Disorders Patient Questionnaire; POMS: Profile of Mood States; MMPI: Minnesota Multiphasic Personality Inventory; HADS: Hospital Anxiety and Depression Scale; GOG PS: Gynaecological Oncology Group Performance Status; BIQ: Body Image Questionnaire.

**Table S2: Outcomes and results Delphi round 1: patients versus healthcare professionals**

| **Round 1** | | | | | | |
| --- | --- | --- | --- | --- | --- | --- |
|  | | **Patients** | | **Healthcare professionals** | |  |
| **Domain** | **Outcome** | **Not important (%)** | **Important (%)** | **Not important (%)** | **Important (%)** | **Decision** |
| **Physiological**  Outcomes relating to neoplasms | Wound healing | 0 | 97 | 0 | 83,3 | Consensus in |
|  | Wound management | 0 | 94,1 | 0 | 58,3 | No consensus |
|  | Vulvar skin changes | 0 | 76,5 | 4,2 | 62,5 | No consensus |
|  | Scar tissue | 2,9 | 82,4 | 4,2 | 75 | Consensus in |
|  | Vaginal discharge | 2,9 | 67,6 | 4,2 | 45,8 | Consensus out |
|  | Vaginal swelling | 0 | 76,5 | 0 | 66,7 | No consensus |
|  | Lymphedema groin area | 0 | 97 | 0 | 87,5 | Consensus in |
|  | Lymphedema legs | 0 | 100 | 0 | 91,7 | Consensus in |
|  | Urinary problems | 0 | 85,3 | 0 | 95,8 | Consensus in |
|  | Urinary incontinence | 0 | 94,1 | 0 | 100 | Consensus in |
|  | Fecal incontinence | 2,9 | 91,1 | 0 | 100 | Consensus in |
|  | Lichen sclerosus | 2,9 | 85,3 | 0 | 83,3 | Consensus in |
|  | Vulvar changes related to sexuality | 11,8 | 73,5 | 0 | 75 | Consensus in |
| **Functioning**  Physical functioning | Role functioning | 0 | 97 | 0 | 95,8 | Consensus in |
|  | Physical functioning | 0 | 97 | 0 | 95,8 | Consensus in |
|  | Cycling | 11,8 | 70,6 | 8.3 | 62,5 | No consensus |
|  | Sitting | 0 | 94,1 | 8,3 | 83,3 | Consensus in |
|  | Wearing clothes | 0 | 82,4 | 12,5 | 79,2 | Consensus in |
|  | Wearing pads or incontinence material | 8,8 | 70,6 | 12,5 | 70,1 | Consensus in |
| **Functioning**  Sexual functioning | Sexual functioning | 5,9 | 61,8 | 0 | 91,7 | No consensus |
|  | Sexual activity without intercourse | 11,8 | 52,9 | 0 | 87,5 | No consensus |
|  | Sexual arousal | 8,8 | 52,9 | 0 | 70,1 | No consensus |
|  | Sexual pleasure | 8,8 | 55,9 | 0 | 70,1 | No consensus |
|  | Masturbation | 26,5 | 38,2 | 0 | 58,3 | Consensus out |
|  | Orgasm | 11,8 | 58,8 | 0 | 50 | Consensus out |
|  | Pain during intercourse | 11,8 | 70,6 | 0 | 83,3 | Consensus in |
|  | Communication about sexuality | 5,9 | 67,6 | 0 | 75 | No consensus |
|  | Sexual satisfaction | 8,8 | 58,8 | 0 | 79,2 | No consensus |
|  | Desire to be sexually active | 14,7 | 61,8 | 0 | 83,3 | No consensus |
|  | Sexual desire | 8,8 | 47 | 0 | 66,7 | Consensus out |
|  | Impact of sexuality on mood | 11,8 | 55,9 | 0 | 66,7 | Consensus out |
| **Functioning**  Social functioning | Social functioning | 5,9 | 85,3 | 0 | 91,7 | Consensus in |
|  | Relationship satisfaction | 8,8 | 73,5 | 0 | 70,8 | Consensus in |
| **Functioning**  Emotional functioning | Impact of wound on mood | 0 | 73,5 | 0 | 66,7 | No consensus |
|  | Emotional functioning | 0 | 88,2 | 0 | 91,7 | Consensus in |
|  | Body image | 0 | 79,4 | 0 | 79,2 | Consensus in |
|  | Feeling comfortable with genital area | 0 | 94,1 | 0 | 58,3 | No consensus |
|  | Concerns about family | 2,9 | 73,5 | 0 | 58,3 | No consensus |
|  | Intimacy | 2,9 | 70,6 | 8,3 | 66,7 | No consensus |
|  | Intimacy satisfaction | 2,9 | 67,6 | 0 | 87,5 | No consensus |
|  | Fear of recurrence | 2,9 | 85,3 | 0 | 77,8 | Consensus in |
|  | Triggers for fear of recurrence | 5,9 | 76,5 | 0 | 62,5 | No consensus |
|  | Psychological stress due to fear of recurrence | 8,8 | 76,5 | 0 | 66,7 | No consensus |
|  | Impact of fear of recurrence on physical functioning | 8,8 | 67,6 | 0 | 70,8 | No consensus |
|  | Confidence in body | 2,9 | 85,3 | 0 | 87,5 | Consensus in |
| **Functioning**  Cognitive functioning | Cognitive functioning | 2,9 | 85,3 | 0 | 75 | Consensus in |
|  | Coping with fear of recurrence | 11,8 | 73,5 | 0 | 70,8 | Consensus in |
| **Global quality of life** | Quality of life | 0 | 88,2 | 0 | 91,7 | Consensus in |
|  | Impact of wound on quality of life | 0 | 82,4 | 0 | 66,7 | No consensus |
| **Delivery of care** | Information provision | 2,9 | 94,1 | 0 | 75 | Consensus in |
|  | Information about disease | 0 | 94,1 | 0 | 70,8 | Consensus in |
|  | Information about treatment | 0 | 94,1 | 0 | 75 | Consensus in |
|  | Information about medical examinations | 0 | 97 | 0 | 66,7 | No consensus |
|  | Information about practical issues | 0 | 88,2 | 0 | 62,5 | No consensus |
|  | Satisfaction with information provision | 5,9 | 82,4 | 0 | 70,7 | Consensus in |

**Table S3: Outcomes and results Delphi round 1: partners versus healthcare professionals**

| **Round 1** | | | | | | |
| --- | --- | --- | --- | --- | --- | --- |
|  | | **Partners** | | **Healthcare professionals** | |  |
| **Domain** | **Outcome** | **Not important (%)** | **Important (%)** | **Not important (%)** | **Important (%)** | **Decision** |
| **Functioning**  Sexual functioning | Sexual functioning | 33,3 | 58,3 | 16,7 | 50 | Consensus out |
|  | Libido | 25 | 41,7 | 16,7 | 45,8 | Consensus out |
|  | Communication about sexuality | 33,3 | 58,3 | 8,3 | 79,2 | No consensus |
|  | Sexual satisfaction | 33,3 | 58,3 | 8,3 | 75 | No consensus |
|  | Importance of sexuality | 33,3 | 66,7 | 8,3 | 62,5 | Consensus out |
|  | Sexual insecurity | 33,3 | 58,3 | 8,3 | 70,1 | No consensus |
|  | Concerns about hurting partner during sexual activity | 16,7 | 66,7 | 12,5 | 70,1 | No consensus |
| **Functioning**  Social functioning | Relationship satisfaction | 8,3 | 58,3 | 4,2 | 70,8 | No consensus |
| **Functioning**  Emotional functioning | Fear of recurrence | 8,3 | 66,7 | 4,2 | 62,5 | Consensus out |
|  | Triggers for fear of recurrence | 8,3 | 50 | 4,2 | 37,5 | Consensus out |
|  | Psychological stress due to fear of recurrence | 8,3 | 66,7 | 4,2 | 37,5 | Consensus out |
|  | Impact of fear of recurrence on physical functioning | 16,7 | 58,3 | 4,2 | 37,5 | Consensus out |
|  | Intimacy | 16,7 | 66,7 | 8,3 | 66,7 | Consensus out |
|  | Intimacy satisfaction | 16,7 | 58,3 | 8,3 | 75 | No consensus |
| **Functioning**  Cognitive functioning | Coping with fear of recurrence | 16,7 | 58,3 | 4,2 | 45,8 | Consensus out |
| **Delivery of care** | Information provision | 0 | 83,3 | 4,2 | 54,2 | No consensus |
|  | Information about disease | 0 | 83,3 | 4,2 | 58,3 | No consensus |
|  | Information about treatment | 0 | 100 | 4,2 | 58,3 | No consensus |
|  | Information about medical examinations | 0 | 91,7 | 4,2 | 54,2 | No consensus |
|  | Information about practical issues | 0 | 91,7 | 8,3 | 54,2 | No consensus |
|  | Satisfaction with information provision | 0 | 75 | 4,2 | 62,5 | No consensus |
|  | Need for emotional support | 8,3 | 58,3 | 4,2 | 50 | Consensus out |
|  | Need for relationship support | 16,7 | 50 | 8,3 | 50 | Consensus out |

**Table S4: Outcomes and results Delphi round 2: patients versus healthcare professionals**

| **Round 2** | | | | | | |
| --- | --- | --- | --- | --- | --- | --- |
|  | | **Patients** | | **Healthcare professionals** | |  |
| **Domain** | **Outcome** | **Not important (%)** | **Important (%)** | **Not important (%)** | **Important (%)** | **Decision** |
| **Physiological**  Outcomes relating to neoplasms | Wound management | 0 | 92,6 | 0 | 66,7 | Included based on patient ratings |
|  | Vulvar skin changes | 0 | 77,8 | 0 | 72,2 | Consensus in |
|  | Vaginal swelling | 0 | 92,6 | 0 | 55,6 | Included based on patient ratings |
| **Functioning**  Physical functioning | Cycling | 14,8 | 81,5 | 0 | 61,1 | Included based on patient ratings |
| **Functioning**  Sexual functioning | Sexual functioning | 7,4 | 55,6 | 0 | 88,9 | No consensus |
|  | Sexual activity without intercourse | 11,1 | 48,1 | 0 | 83,3 | No consensus |
|  | Sexual arousal | 8,3 | 54,1 | 0 | 70,6 | No consensus |
|  | Sexual pleasure | 11,1 | 59,3 | 0 | 72,2 | No consensus |
|  | Communication about sexuality | 3,7 | 74,1 | 0 | 61,1 | Included based on patient ratings |
|  | Desire to be sexually active | 14,5 | 63 | 0 | 83,3 | No consensus |
|  | Sexual satisfaction | 11,1 | 55,6 | 0 | 77,8 | No consensus |
| **Functioning**  Emotional functioning | Impact of wound on mood | 3,7 | 81,5 | 0 | 61,1 | Included based on patient ratings |
|  | Feeling comfortable with genital area | 0 | 88,9 | 0 | 66,7 | Included based on patient ratings |
|  | Concerns about family | 3,7 | 70,4 | 0 | 50 | Included based on patient ratings |
|  | Intimacy | 3,7 | 66,7 | 0 | 83,3 | No consensus |
|  | Intimacy satisfaction | 3,7 | 66,7 | 0 | 83,3 | No consensus |
|  | Triggers for fear of recurrence | 7,4 | 74,1 | 0 | 66,7 | Included based on patient ratings |
|  | Psychological stress due to fear of recurrence | 3,7 | 77,8 | 0 | 66,7 | Included based on patient ratings |
|  | Impact of fear of recurrence on physical functioning | 8,8 | 67,6 | 0 | 70,8 | No consensus |
| **Global quality of life** | Impact of wound on quality of life | 0 | 88,9 | 0 | 83,3 | Consensus in |
| **Delivery of care** | Information about medical examinations | 0 | 92,6 | 0 | 55,6 | Included based on patient ratings |
|  | Information about practical issues | 0 | 81,4 | 0 | 50 | Included based on patient ratings |

**Table S5: Outcomes and results Delphi round 2: partners versus healthcare professionals**

| **Round 2** | | | | | | |
| --- | --- | --- | --- | --- | --- | --- |
|  | | **Partners** | | **Healthcare professionals** | |  |
| **Domain** | **Outcome** | **Not important (%)** | **Important (%)** | **Not important (%)** | **Important (%)** | **Decision** |
| **Functioning**  Sexual functioning | Communication about sexuality | 33,3 | 66,7 | 11,1 | 72,2 | No consensus |
|  | Sexual satisfaction | 22,2 | 55,6 | 5,5 | 77,8 | No consensus |
|  | Sexual insecurity | 33,3 | 66,7 | 16,7 | 66,7 | Consensus out |
|  | Concerns about hurting partner during sexual activity | 22,2 | 55,6 | 22,2 | 66,7 | Consensus out |
| **Functioning**  Social functioning | Relationship satisfaction | 11,1 | 55,6 | 5,5 | 72,2 | No consensus |
| **Functioning**  Emotional functioning | Intimacy satisfaction | 22,2 | 55,6 | 5,5 | 77,8 | No consensus |
| **Delivery of care** | Information provision | 0 | 77,8 | 0 | 66,7 | Included based on partner ratings |
|  | Information about disease | 0 | 100 | 0 | 72,2 | Consensus in |
|  | Information about treatment | 0 | 100 | 0 | 72,2 | Consensus in |
|  | Information about medical examinations | 0 | 77,8 | 0 | 66,7 | Included based on partner ratings |
|  | Information about practical issues | 0 | 77,8 | 5,5 | 61,1 | Included based on partner ratings |
|  | Satisfaction with information provision | 0 | 77,8 | 0 | 66,7 | Included based on partner ratings |

**Table S6: Outcomes and results consensus meeting patients versus healthcare professionals**

| **Consensus meeting** | | | | |
| --- | --- | --- | --- | --- |
| **Domain** | **Outcome** | **Not important (%)** | **Important (%)** | **Decision** |
| **Physiological**  Outcomes relating to neoplasms | Vaginal odor | 21 | 79 | Consensus in |
|  | Change in sensation of the genital area | 7 | 93 | Consensus in |
| **Functioning**  Sexual functioning | Sexual functioning | 21 | 79 | Consensus in |
|  | Sexual activity without intercourse | 43 | 57 | Consensus out |
|  | Sexual arousal | 36 | 64 | Consensus out |
|  | Sexual pleasure | 36 | 64 | Consensus out |
|  | Desire to be sexually active | 14 | 86 | Consensus in |
|  | Sexual satisfaction | 14 | 86 | Consensus in |
| **Functioning**  Social functioning | Impact on entering (new) relationship(s) | 43 | 57 | Consensus out |
| **Functioning**  Emotional functioning | Intimacy | 21 | 79 | Consensus in |
|  | Intimacy satisfaction | 29 | 71 | Consensus in |
|  | Impact of fear of recurrence on physical functioning | 14 | 86 | Consensus in |
